# Mini-clusters with mean probabilities for identifying effective siRNAs

**DOI:** 10.1186/1756-0500-5-512

**Published:** 2012-09-18

**Authors:** Jia Xingang, Zuhong Lu, Qiuhong Han

**Affiliations:** 1 Department of Mathematics, Southeast University, Nanjing 210096, PR China; 2 State Key Laboratory of Bioelectronics, School of Biological Science and Medical Engineering, Southeast University, Nanjing 210096, PR China; 3 Department of Mathematics, Nanjing Forestry University, Nanjing 210037, PR China

## Abstract

**Background:**

The distinction between the effective siRNAs and the ineffective ones is in high demand for gene knockout technology. To design effective siRNAs, many approaches have been proposed. Those approaches attempt to classify the siRNAs into effective and ineffective classes but they are difficult to decide the boundary between these two classes.

**Findings:**

Here, we try to split effective and ineffective siRNAs into many smaller subclasses by RMP-MiC(the relative mean probabilities of siRNAs with the mini-clusters algorithm). The relative mean probabilities of siRNAs are the modified arithmetic mean value of three probabilities, which come from three Markov chain of effective siRNAs. The mini-clusters algorithm is a modified version of micro-cluster algorithm.

**Conclusions:**

When the RMP-MiC was applied to the experimental siRNAs, the result shows that all effective siRNAs can be identified correctly, and no more than 9% ineffective siRNAs are misidentified as effective ones. We observed that the efficiency of those misidentified ineffective siRNAs exceed 70%, which is very closed to the used efficiency threshold. From the analysis of the siRNAs data, we suggest that the mini-clusters algorithm with relative mean probabilities can provide new insights to the applications for distinguishing effective siRNAs from ineffective ones.

## Findings

RNA interference (RNAi) is a cellular process for sequence specific destruction of mRNA [1]. The broad mechanistic details for the pathway have been largely characterized. Long double-stranded RNAs duplex or hairpin precursors are cleaved into small interfering RNAs (siRNAs) by the ribonuclease III enzyme Dicer. The typical siRNAs have a 19-nucleotide paired region followed by a 2-nucleotide 3’ overhang [2]. The siRNAs are used to initiate RNAi [3-6]. Therefore, the distinguishing the effective siRNAs from the ineffective ones is in high demand for gene knockout technology. In order to design effective siRNAs, many computational approaches have been proposed [7-20]. Some approaches focus on finding the common features of effective siRNAs, though they initially and intuitively provide guidelines for siRNAs design, are far from satisfied due to low sensitivity and specificity [8,18]. The other approaches are motivated by statistical learning theory, attempt to classify the siRNAs into effective and ineffective classes. Although those two-class classifiers provide a promising way to screen potentially effective siRNAs, it is difficult to decide the boundary between the two classes.

Here, we use the set of effective siRNAs to estimate distributions of three Markov chains, where the order of three Markov chain are 1, 2 and 3, respectively. Each siRNA obtain three probabilities from the distributions of three Markov chains. Based on three probabilities of siRNAs, we introduce a robust feature of siRNAs, the relative mean probabilities, which is the modified arithmetic mean value of these three probabilities. It should be noticed that the siRNAs with similar relative mean probabilities have same efficacy(effective/ineffective) usually, most relative mean probabilities of effective siRNAs exceed most ineffective ones. However, there is no clear boundary between these two classes, so we give up the attempt of dichotomy. We try to split these two classes into many smaller effective or ineffective subclasses, respectively. Thus, we distinguish effective siRNAs from the ineffective ones by a mini-clusters algorithm, which adopted from [21](see Materials and methods). By RMP-MiC(the relative mean probabilities with the mini-clusters), all effective siRNAs can be identified correctly, and no more than than 9% ineffective siRNAs are misidentified as effective siRNAs. We observed that the efficiency of those misidentified ineffective siRNAs exceed 70%, which is very closed to the used efficiency threshold.

## Methods

### Estimating distributions of siRNAs

The siRNAs can be represented as an 19-tuple of vector. *x*_*i *_= (*x*_*i*1_,*x*_*i*2_,···,*x*_*i*19_) is the *i*-th siRNA where *x*_*ij *_represents its *j*-th nucleotide. Effective siRNAs are used to estimate *Q*_*h*_, where *Q*_*h *_is distribution of a *h*-order Markov chain, *h* equals 1, 2 and 3, respectively. *Q*_*h *_(*i*) is probability of the *i*-th siRNA in *Q*_*h*_. We use *Q*_*h *_(*i*)(*h *= 1,2,3) to construct *Q*_4 _(*i*), where

(1) $$Q_{4}(i)=\frac{Q_{1}(i)+Q_{2}(i)+Q3(i)}{\left\lceil Q_{1}(i)\rceil +\lceil Q_{2}(i)\rceil +\lceil Q_{3}(i)\right\rceil },$$

(2) $$Q_{h}(i)=\text{Pr}(x_{i1}\cdot \cdot \cdot x_{\text{ih}})\overset{19}{\underset{s=h+1}{\prod }}q_{h}(\text{ij}),$$

(3) $$q_{h}(\mathrm{ij})=\text{Pr}(x_{\text{is}}|x_{i(j-h)},\cdot \cdot \cdot \;,x_{i(j-1)}).$$

If *Q*_*h *_(*i*) exceed zero, ⌈*Q*_*h *_(*i*)⌉ is 1, otherwise ⌈*Q*_*h *_(*i*) ⌉ is zero. *Q*_4 _(*i*) name as relative mean probabilities of *x*_*i*_. It can be noticed that the siRNAs with similar relative mean probabilities have alike efficiency usually.

### Mini-clusters algorithm

Based on the relative mean probabilities of siRNAs, we distinguish effective siRNAs from the ineffective ones by a mini-clusters algorithm, which adopted from [21], a commonly used micro-cluster algorithm. It is sketched as below.

Define the distance between *i*-th and *j*-th siRNAs as

(4) $$d_{h}(i,j)=\sqrt{{\left(Q_{h}(i)-Q_{h}(j)\right)}^{2}}.$$

We put the closest two elements in a cluster. In subsequent steps, we examine the two closest elements not already in a cluster. If either or both of these are closer to some element within a cluster, we put each element in the cluster to which it is closest, otherwise, we form a new cluster. Repeat this step until all siRNAs have been put into a mini-cluster.

For the siRNAs in testing set, we consider that their efficiency are unknown. In the process of testing the sensitivity and specificity, a mini-cluster is considered as effective if it has an effective siRNAs, and be considered as ineffective if all siRNAs are ineffective, otherwise its efficacy is uncertain. We denote effective, ineffective and uncertain mini-clusters as

(5) $$A_{1},A_{2},\cdot \cdot \cdot \;,A_{u};\;\;\;A_{u+1},A_{u+2},\cdot \cdot \cdot \;,A_{a};\;\;\;B_{1},B_{2},\cdot \cdot \cdot \;,B_{b};$$

respectively. Define the distance of *A*_*i *_and *B*_*j *_as

(6) $$d_{h}(A_{i},B_{j})=\underset{u\in A_{i},v\in B_{j}}{\text{min}}d_{h}^{s}(u,v).$$

If

(7) $$d_{h}(A_{i1},B_{j})=\underset{i=1,2\cdot \cdot \cdot \;,s}{\text{min}}d_{h}(A_{i},B_{j}),$$

the efficacy of *B*_*j*_ is regarded as that of *A*_*i*1_. In other words, each uncertain mini-cluster is merged into the nearest determined ones.

## Availability

### Testing the performance of mini-clusters

To test the performance of RMP-MiC, it was firstly applied to a simulation data. The sequences of simulation data set belong to two groups *X* and *Y *, each of them contains 5 nucleotides. In order to simplify the problem, we assume the nucleotides are generated from different 1-order Markov chain, that is, the relative mean probabilities of sequences equal the probabilities of their 1-order Markov chain. For *X*, the probabilities of U base and C base at position 1 are 0.75 and 0.25, conditional probabilities of position 2 are

(8) Pr(A|U)=0.75,Pr(U|U)=0.25,Pr(G|C)=1

and others are zero. At 3-5 position, we assume that all conditional probabilities are 0.25. For each sequence of *Y *, we assume that ’U’ base at position 1 and ’A’ base at position 5 or ’C’ base at position 1 and ’G’ base at position 5 can not appear at the same time, nucleotides are random at other positions. An illustrative example within the simulation data is shown in Table 1, which consists of 17 sequences. These 17 sequences belong to two groups *X* and *Y *. The two groups are of size 10 and 7, respectively. The relative mean probabilities of these 17 sequences are shown in Table 1. For comparison, we also applied K-mean with Euclidean to cluster all sequences into 2 cluster, where the distance between two sequences are Euclidean distance of their mean probabilities. The clustering results by two methods are shown in Table 1.

**Table 1:** List of simulation data and clustering results by two algorithm

								**Results**	
**Group *X***	**Sequences**	***P***_**1**_	***P***_**2**_	***P***_**3**_	***P***_**4**_	***P***_**5**_	***Q*(4)**	**RMP-MiC**	**K-mean**
a1	UAAUC	0.75	0.75	0.25	0.25	0.25	0.0088	1	1
a2	UACCG	0.75	0.75	0.25	0.25	0.25	0.0088	1	1
a3	UAGAA	0.75	0.75	0.25	0.25	0.25	0.0088	1	1
a4	UUCCG	0.75	0.25	0.25	0.25	0.25	0.0029	2	2
a5	UUGAA	0.75	0.25	0.25	0.25	0.25	0.0029	2	2
a6	UUUGU	0.75	0.25	0.25	0.25	0.25	0.0029	2	2
a7	CGAUC	0.25	1	0.25	0.25	0.25	0.0039	3	2
a8	CGCCG	0.25	1	0.25	0.25	0.25	0.0039	3	2
a9	CGGAA	0.25	1	0.25	0.25	0.25	0.0039	3	2
a10	CGUGU	0.25	1	0.25	0.25	0.25	0.0039	3	2
**Group *Y***									
b1	AACGA	0	0	0.25	0.25	0.25	0	4	2
b2	AUGGA	0	0	0.25	0.25	0.25	0	4	2
b3	UCAGC	0.75	0	0.25	0.25	0.25	0	4	2
b4	UGUUC	0.75	0	0.25	0.25	0.25	0	4	2
b5	UCCUG	0.75	0	0.25	0.25	0.25	0	4	2
b6	CCAAA	0.25	0	0.25	0.25	0.25	0	4	2
b7	CCUAC	0.25	0	0.25	0.25	0.25	0	4	2

*P*_1 _is the probabilities of the leftmost nucleotides. *P*_*i *_(*i *= 2,3,4,5) is conditional probabilities of the *i*-th position. *Q*(4) is the the relative mean probabilities of sequences.

In Table 1, RMP-MiC grouped these 17 sequences into 4 mini-clusters, sequences of each mini-clusters come from the same group. The Euclidean algorithm were clusters 7 sequences of cluster 1 incorrectly grouped in cluster 2. The reason may be that Euclidean distance takes the difference between data points directly, it may be overly sensitive to the magnitude of changes To further test these methods, we applied it to a larger data set containing 1,000 samples. Results were similar to those observed for the smaller data set(data not shown).

### Identifying results of the experimental siRNAs

The data set can be downloaded from http://www.bioinf.seu.edu.cn/siRNA/Supplementary/index.htm. It collects 3589 experimental validated siRNAs from 9 publications [7,10-12,22-26]. The efficiency threshold of siRNA to be effective is 80%. According to this threshold, the data set has 582 effective siRNAs and 3007 ineffective siRNAs.

To validate the performance of *Q*_4 _(*i*) with mini-clusters, we apply them to data set of experimental siRNAs, where *Q*_4 _(*i*) are estimated by all effective siRNAs. The identifying results are summarized in Table 2. In fact, all effective siRNAs are correctly identified and only 264 ineffective siRNAs are misidentified into effective siRNAs by *Q*_4 _(*i*) with mini-cluster. It should be noticed that when ineffective siRNAs are misidentified into effective siRNAs, its efficiency exceeds 70% mostly.

**Table 2:** The identifying results of siRNAs by five different algorithms

**Algorithm**	**Feature**	**Total**	**Sensitivity(%)**	**Specificity(%)**
Mini-cluster	*Q*_1_(*i*)	1192	1	48.83
Mini-cluster	*Q*_2_(*i*)	1116	1	52.15
Mini-cluster	*Q*_3_(*i*)	682	1	85.34
Mini-cluster	*Q*_4_(*i*)	846	1	68.79
K-means	*Q*_4_(*i*)	1588	1	36.65

The total number is the number of the identified effective siRNAs. Sensitivity, the number of effective siRNAs/582. Specificity, the number of effective siRNAs/total number of cluster members.

For comparison, we applied the $Q_{h}^{s}(i)(h=1,2,3)$ with mini-clusters to the same data. The K-mean with Euclidean was also applied to cluster all sequences into 2 cluster, where the distance between two sequences are Euclidean distance of their *Q*_4 _(*i*), the number of clusters is the same as the number of mini-clusters of *Q*_4 _(*i*). The results are also summarized in Table 2. These results show that all effective siRNAs are correctly identified and 610, 534 and 100 ineffective siRNAs are misidentified with effective siRNAs by *Q*_1 _(*i*), *Q*_2 _(*i*) and *Q*_3 _(*i*) with mini-cluster, respectively.

For comparison, The K-mean with Euclidean was also applied to cluster all sequences into 2 cluster, where the distance between two sequences are Euclidean distance of their *Q*_4 _(*i*), the number of clusters is the same as the number of mini-clusters of *Q*_4 _(*i*). The results are also summarized in Table 2. These result shows that all effective siRNAs are correctly identified but 1006 ineffective siRNAs are misidentified with effective siRNAs.

To test the sensitivity and specificity of *Q*_4 _(*i*) with mini-clusters, 80% effective siRNAs are chosen as training data set. The siRNAs of training data set are used to estimate *Q*_4 _(*i*). To assure each siRNA may be in test set, we construct 1,000 different training data set. The results show that only 13 effective siRNAs are incorrectly identified and 516 ineffective siRNAs are misidentified with effective siRNAs , where the number of the misidentified effective and ineffective siRNAs are the mean values acquired from averaging across each training set. The result shows that *Q*_4_(*i*) with mini-clusters is reliable for identifying effective siRNAs. However, when we use *Q*_3_(*i*) to substitute *Q*_4_(*i*), only 18% effective siRNAs of training data set can identify correctly. The reason may be that many *Q*_3_(*i*) of effective siRNAs of training data set become zero. It can result in which these effective siRNAs are misidentified to ineffective siRNAs. However, even if *Q*_3_(*i*) of these effective siRNAs are zero but their *Q*_1_(*i*) and *Q*_2_(*i*) may be very large, so their *Q*_4_(*i*) are also different with ineffective siRNAs. Thus, they may construct new mini-clusters or enter into effective mini-clusters.

Secondly, we randomly generate 1,0000 simulation siRNAs. A new data set of siRNAs are formed by these 1,0000 simulation siRNAs and 3587 experimental siRNAs. By *Q*_4_(*i*) with mini-clusters, these 1,3587 siRNAs are put into different mini-clusters, where 1587 simulation siRNAs are put into effective mini-clusters, *Q*_4_(*i*) are estimated by all effective experimental siRNAs. The efficiency of these 1587 simulation siRNAs are de novo validated by a web-server RFRCDB-siRNA [27], which is available at http://www. bioinf.seu.edu.cn/siRNA/index.htm. By the web-server, 1536 simulation siRNAs are identified as effective. The result shows that effective siRNAs should have specific features at some positions, and *Q*_4_(*i*) can incarnate these specific features.

### Identifying results of the shRNAs

To systematically analyze the interplay between nucleotide composition, shRNA processing, and biologic activity, Christof Fellmann *et al* transduced the entire Sensor library into human HEK293T and chicken ERC cells, generated and quantified small RNA libraries designed to represent shRNA intermediates after major biogenesis steps, which contains 18,720 shRNAs [28]. The efficiency threshold of shRNA to be effective is that its score exceed 10. According to this threshold, the data set has 453 effective siRNAs and 18267 ineffective siRNAs. The data set of shRNAs can be downloaded from: http://www.ncbi.nlm.nih.gov/ pmc/articles/PMC3130540/?tool=pubmed.

To validate the performance of *Q*_4_(*i*) with mini-clusters to distinguish effective shRNAs, it is applied to data set of shRNAs, where *Q*_4_(*i*) are estimated by all effective shRNAs. The identifying results shows that all effective shRNAs are correctly identified and only 1446 ineffective shRNAs are misidentified into effective shRNAs by *Q*_4_(*i*) with mini-cluster. It should be noticed that when ineffective shRNAs are misidentified into effective shRNAs, their efficiency are very closed to the effective threshold.

### Comparison to existing design algorithms

To compare our results to existing siRNA-based design tools, we obtained the top predictions for transcripts using three different algorithms [17-19] and compared them to the 50 highest scoring Sensor-derived shRNAs for gene. Strikingly, exceed 70% of scoring shRNAs were not identified in the top 50 predictions of any algorithm. While such false negatives, in principle, may have little practical significance, the majority of algorithm-predicted shRNAs did not score in the Sensor assay, closely resembling their low validation rate in empirical testing. Together, these results demonstrate that siRNA algorithms are poor at predicting potent shRNAs [29] and underscore the value of the Sensor approach.

## Requirements

Since effective siRNAs have specific nucleotides at some position, it is reasonable to use relative mean probabilities as their feature indicator. However, effective siRNAs may have different relative mean probabilities, but the mini-clusters algorithm place siRNAs with similar relative mean probabilities in the same mini-clusters.

In fact, relative mean probabilities can be viewed as specific probabilities of siRNAs, so the absolute value of their logarithm can be regarded as entropies of siRNAs. Since siRNAs with similar relative mean probabilities are in the same mini-clusters, the deviance of efficiency of siRNAs can be regarded as the difference in their entropies.

## Conclusions

From the analysis of the siRNAs data, we demonstrate that mini-clusters algorithm using *Q*_4_(*i*) are appropriate for analyzing siRNAs data. Its success indicates that an effective algorithms for analyzing biological data must be based on an understanding of the biological nature of the experimental data.

## Competing interests

The authors declare that they have no competing interests.

## Authors’ contributions

Conceived and designed the experiments: JX and ZL. Performed the experiments: QH. Analyzed the data: JX. Wrote the paper: JX. All authors read and approved the final manuscript.
